# Effect of Pulsed Electric Fields on the Shelf Stability and Sensory Acceptability of Osmotically Dehydrated Spinach: A Mathematical Modeling Approach

**DOI:** 10.3390/foods13091410

**Published:** 2024-05-03

**Authors:** George Dimopoulos, Alexandros Katsimichas, Konstantinos Balachtsis, Efimia Dermesonlouoglou, Petros Taoukis

**Affiliations:** Laboratory of Food Chemistry and Technology, School of Chemical Engineering, National Technical University of Athens (NTUA), 15780 Athens, Greece; gdimop@chemeng.ntua.gr (G.D.); alkats@chemeng.ntua.gr (A.K.); kbalachtsis@hotmail.gr (K.B.); efider@chemeng.ntua.gr (E.D.)

**Keywords:** *Spinacia oleracea*, leafy vegetables, nonthermal processing, osmotic dehydration, pulsed electric fields, shelf-life study

## Abstract

This study focused on the osmotic dehydration (OD) of ready-to-eat spinach leaves combined with the pulsed electric field (PEF) pre-treatment. Untreated and PEF-treated (0.6 kV/cm, 0–200 pulses) spinach leaves were osmotically dehydrated at room temperature for up to 120 min. The application of PEF (0.6 kV/20 pulses) prior to OD (60% glycerol, 25 °C, 60 min) lowered water activity (*a_w_* = 0.891) while achieving satisfactory product acceptability (total sensory hedonic scoring of 8). During the storage of the product (at 4, 8, 12, and 20 °C for up to 30 d), a significant reduction in total microbial count evolution was observed (9.7 *logCFU*/g for the untreated samples vs. 5.1 *logCFU*/g for the PEF-OD-treated samples after 13 d of storage at 4 °C). The selection of these PEF and OD treatment conditions enabled the extension of the product shelf life by up to 33 d under chilled storage. Osmotically treated spinach could find application in ready-to-eat salad products with an extended shelf life, which is currently not possible due to the high perishability of the specific plant tissue.

## 1. Introduction

Spinach (*Spinacia oleracea* L.) is a highly valued leafy green popular for its nutritional diversity, including bioactive compounds such as carotenoids, chlorophylls, flavonoids, and phenolic acids, as well as its versatility and sensory appeal [1]. Compared to cabbage and lettuce, it contains two and four times more free phenolics, respectively [2]. It is widely consumed in ready-to-eat (RTE) salads, and its shelf-life extension is of high commercial interest since its high perishability makes handling, shipping, and storage expensive and complex and restricts use and marketability. Most of the scientific studies involving spinach investigate the effect of storage conditions (e.g., storage temperature), packaging (e.g., modified atmosphere), and industrial processing (e.g., drying) on the product quality characteristics (e.g., visual quality, nutritional value) [1,2,3,4,5].

Osmotic dehydration (OD) is a food processing technique that involves partially removing water from plant tissues (mostly fruits and vegetables) by immersing them in a hypertonic solution. The higher osmotic pressure of the solution drives water out of the tissue. Simultaneously, solutes form in the solution and diffuse into the tissue. This technique aims to preserve or even improve product quality (such as color and flavor retention, superior sensory characteristics, and enzymatic browning inhibition). Factors affecting osmotic dehydration include the temperature of the osmotic solution, the concentration of the osmotic solution, the type of osmotic agent used, the process time, the ratio of food material to osmotic solution, and the geometry of the food material [6,7,8]. The primary purpose of osmotic dehydration is to reduce the moisture content of foods before further processing (such as drying). At the same time, the selective enrichment of the osmosed tissue with the solutes of the osmotic solution can produce a modified food product with the desired quality attributes [7]. The potential use of novel osmotic solutes (such as high-value-added ingredients and food industry by-products) has been more recently investigated. The enhancement of mass transfer rates (water and solutes) with the application of suitable pre-treatment methods to achieve shorter osmotic dehydration times has also been studied [9,10].

The use of the pulsed electric field (PEF) treatment has been proposed as a pre-treatment method for dehydration processes, both traditional (air drying) and non-traditional (osmotic dehydration) [10,11,12]. Pulsed electric fields is a nonthermal processing method that utilizes short pulses of electricity to achieve microbial inactivation while minimizing detrimental effects on food product quality. It involves applying high-voltage pulses to liquid or semi-liquid foods placed between two electrodes. The electric field induces electroporation, creating pores in the cell membrane and walls. This increases cell permeability, allowing intercellular material, primarily water, to move to the extracellular matrix [13]. While the increase in water loss greatly speeds up the drying process, it can also lead to a loss of cell turgor pressure, resulting in tissue softening and, consequently, texture deterioration in plant tissues [14].

Pulsed electric fields do not modify the content of phenolic compounds and vitamin C in fruit/vegetable juices. Studies reported an increase in phenolic compounds as well as vitamin C content owing to the mass transfer induced by electroporation within the cells into the aqueous medium of the juice, making them more available in the liquid medium and thus quantifiable by detection methodologies [15,16]. The increase in phenolic compounds is associated with an increase in antioxidant capacity [17,18]. The application of PEF do not compromise the content of anthocyanins and flavonoids in fruit and vegetable juices, as reported. Thus, the bioactive properties and sensory characteristics, such as color, are preserved in PEF-treated samples [19]. Some studies suggested a decrease in the above-mentioned compounds when the electric field strength values exceeded 19 kV/cm [19,20]. An increase in or possible release of minerals and salts has also been observed in PEF-treated food products (such as Ni, Cu, and Cr in chicken and beef and Ca in pumpkin) [21]. The positive effect of PEF on the biochemical, functional, and nutritional characteristics of OD-treated food products has been reported. The combination of OD and PEF has been proven to be an effective processing step in the production of intermediate-moisture products or a pre-processing step in the production of dried or frozen products [22,23]. An increase in antioxidant capacity and activity as well as vitamin C content in OD-treated fruits and vegetables after PEF pre-treatment has been observed [22,23,24].

The PEF effect on dried spinach quality (color and vitamin C content) and drying process parameters (rate) have been studied [25]. According to Yamakage et al. (2021), the drying rate of the PEF-processed samples (2.8 kV/cm of the electric field strength and 27.1 kJ/kg of the specific input energy) increased compared to the unprocessed and hot water samples [25]. Furthermore, degradation of the surface color and vitamin C (L-ascorbic acid) in the PEF-processed samples was significantly inhibited compared to the hot water pre-processing step. The results showed that PEF could be applied to reduce drying time and produce dried spinach of superior quality. The PEF effect on protecting color in spinach puree [26] and spinach extract [27] has also been reported.

The effect of pulsed electric fields and/or osmotic dehydration on the quality preservation and shelf-life extension of salad leaves, such as spinach leaves, has not been investigated. Osmotic dehydration has been employed to improve the quality and extend the shelf life of fruit and vegetable cuts, but not fresh leafy vegetables. The impregnation of spinach leaves with cryoprotectants using a combination of vacuum impregnation (VI) and PEF has been so far proposed by Dymek et al. (2016) as a method of improving their freezing tolerance and, consequently, their general quality after thawing [28].

This study aims to evaluate the effect of PEF treatment conditions on the quality parameters, both sensory and physicochemical, and microbiological stability of osmotically dehydrated spinach leaves (during cold storage). A preliminary screening of PEF treatment conditions (varying specific energy input through the number of pulses and the electric field strength) was implemented to identify the suitable treatment window. Successfully dehydrated spinach could potentially find application in ready-to-eat salad products with an extended shelf life, which is currently not possible due to the high perishability of the specific plant tissue.

## 2. Materials and Methods

### 2.1. Raw Material Selection

Commercially available ready-to-eat (RTE) spinach leaves were supplied by a leading processor (Barbastathis, Athens, Greece) with a 10-day “use by” date. Treatments of spinach leaves were performed within 1 day of supply.

### 2.2. Selection of OD Treatment Conditions

For the OD of spinach, the osmotic medium was prepared in tap water and contained glycerol, 1.5% calcium chloride, 1% sodium chloride, 2.5% vinegar (8% acetic acid), and 0.05% *w/w* sodium sulfite. Two different glycerol contents of the medium were studied, namely 50 and 60% *w/w*. Glycerol acts as a humectant and plasticizer. Sodium chloride enhances mass transfer, and calcium chloride significantly contributes to the preservation of plant tissue texture. Vinegar serves as a flavoring agent and contributes to the reduction in the treated food’s pH [14]. Sulfites act as inhibitors of enzymatic browning, which can be enhanced when cellular disruption pre-treatments such as PEF are applied. For all treatments, the duration of OD was studied up to 120 min at room temperature.

### 2.3. Selection of PEF Pre-Treatment Conditions

According to the literature [28,29,30], electric field strengths as low as 0.5 kV/cm are sufficient to cause cellular disruption adequate for effective dehydration. Spinach leaves were treated in water (tap) (electrical conductivity 800 μS/cm) at different electric field strengths from 0.6 to 2.2 kV/cm and number of pulses (0 (untreated)–200 pulses). For the selection of the suitable PEF condition (number of pulses at different electric fields), sensory evaluation (comprising perceived turgor intensity, toughness, and total sensory acceptance) was carried out. Pulsed electric fields were conducted (Elcrack-5 kW, DIL, Quackenbrück, Germany) in a stainless-steel treatment chamber with an electrode spacing of 8 cm and a total volume of 300 mL. The pulse width and pulse frequency were fixed at 15 μs and 20 Hz, respectively.

### 2.4. OD Treatment of Non- and PEF-Pre-Treated Samples

**Scheme 1:**
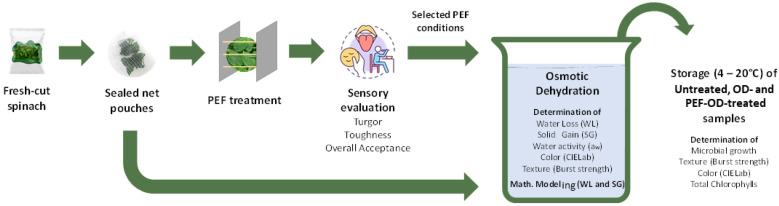
Experimental procedure for PEF-OD treatment of spinach leaves.

Non-pre-treated and selected PEF-pre-treated spinach leaves were osmotically dehydrated using two different media, as described in Section 2.2. Freshly cut spinach leaves were weighed (approximately 5 g), enveloped in a fine mesh net, and placed in 2 L glass beakers containing the osmotic medium under gentle agitation at 25 °C. The solid-to-liquid ratio was fixed at 1:20. Samples were taken at 0, 20, 40, 60, 90, and 120 min for determination of water activity measurement and mass transfer calculations (water loss and solid gain) (Scheme 1, Table 1).

#### 2.4.1. Mathematical Modeling of Water Loss and Solid Gain

Water loss (WL) and solid gain (SG) represent the mass of initial water that is lost during dehydration and the mass of solids taken up by the sample during osmotic dehydration, respectively, calculated from the following equations (respectively, Equations (1) and (2)):

(1) $$WL=\frac{m_{0}-m_{0}\cdot DW_{wb}-(m_{wet}-m_{dry})}{m_{0}\cdot DW_{wb}}$$

(2) $$SG=\frac{m_{dry}-DW_{wb}\cdot m_{0}}{DW_{wb}\cdot m_{0}}$$

where *m*_0_ is the sample initial wet weight before submersion in the osmotic medium, *DW_wb_* is the dry weight of the untreated sample in g water/g sample wet base, *m_wet_* is the wet weight of the treated sample in g, and *m_dry_* is the dry weight of the treated sample in g.

Water loss and solid gain of spinach leaves were then modeled using the penetration model, where *WL/SG* was assumed to be proportional to the square root of the contact time [31,32], as follows:

(3) $$WL\;or\;SG=k_{X}\;\cdot \sqrt{t}$$

where *WL* is in $\frac{gwater}{gDW}$ or *SG* is in $\frac{gsolid}{gDW}$ at time *t*; *k_X_* is the water loss constant (*k_WL_*) in gwatergDW·s12 or the solid gain constant (*k_SG_*) in gsolidsgDW·s12; and *t* is the osmotic dehydration time in min. The water loss and solid gain constants were determined by nonlinear regression of the experimental data (SPSS 19, IBM, Armonk, NY, USA).

#### 2.4.2. Determination of Physicochemical Parameters

For each sample, water content and dry matter during osmotic dehydration were determined gravimetrically after drying at 105 °C for 24 h [33,34]. Water activity was measured using a water activity meter (Rotronic AM3 Hygrometer, Bassersdorf, Switzerland). pH values were measured using a benchtop AMEL 338 (AMEL Electrochemistry S.r.l., Milano, Italy) pH meter in the homogenized spinach samples.

#### 2.4.3. Determination of Leaf Burst Strength

Burst strength of leafy vegetables corresponds to the maximum force required to puncture a taut leaf. It is a measure of how “tough” a leaf is and may be affected by treatments that alter the cell structure and partitioning of water within the cells. Burst strength was determined using a TA.XT2i Stable Micro Systems texture analyzer (Stable Micro Systems, Godalming, Surrey, UK), which was connected to a computer equipped with the appropriate software. For each sample, a measurement was carried out with a suitable strain at a representative point of the spinach sample. The Film Support Rig (HDP/FSR) (Stable Micro Systems, Godalming, Surrey, UK) was used to measure the burst strength, which consists of two hole-bearing plates and a punching rod. For the measurement, a spinach leaf was fixed between the perforated plates and pierced by the descending probe of the analyzer. The maximum force recorded during the analysis is defined as the punching force.

#### 2.4.4. Color Measurement

The color was measured on the surface of the spinach leaf at three points on at least five replicates using an Xrite-i1 portable digital colorimeter (Gretag-Macbeth, Grand Rapids, MI, USA) and expressed in the CIE-Lab scale, where L-value: lightness; a-value: redness and greenness; and b-value: yellowness and blueness (CIE 1978) [35]. Color was measured at three points on at least five replicates. Color difference between treated and untreated samples was expressed using the total color difference Δ*E* [36], given by the following equation (Equation (4)):

(4) $$\Delta E=\sqrt{{\left(a-a_{0}\right)}^{2}+{\left(b-b_{0}\right)}^{2}+{\left(L-L_{0}\right)}^{2}}$$

where *L*, *a*, *b* are the measured CIELab color parameters and *L*_0_, *a*_0_, *b*_0_ are the measured color parameters of a reference sample.

#### 2.4.5. Sensory Analysis

For all non-pre-treated and pre-treated spinach samples, sensory analysis was carried out by 8 panelists during their cold storage. Spinach leaves were scored by the panelists for the following properties: intensity of green color, perceived color luminosity, perceived turgor intensity, perceived tissue elasticity, perceived off-flavor intensity, and overall sensory acceptance on a hedonic scale of 1–9. The sensory rejection score was fixed at 5 [37]. Ready-to-eat (RTE) pre-cut and washed spinach leaves were used; the additional PEF and/or OD treatments were expected to decrease any risk that would theoretically be of concern in the ready-for-consumption plant material. Given the hygienic handling and OD processing of the samples, testing by the panelists was of lesser risk than the untreated commercially available RTE spinach leaves.

Only adults participated in the recruitment for the sensory team. Participation in the tests and assessments was voluntary. Informed written consent was obtained from the participants in the sensory evaluation study. Each of them could withdraw their consent without providing any justification. All participants obtained a detailed description of the test and were informed about the food samples that would be assessed. Each of the participants was obliged to report any indispositions or allergies, and if such was the case, the subject did not participate in the tests.

#### 2.4.6. Determination of Microbial Load

For the determination of microbial growth, the method of agar-plate colony enumeration was used. For each sample, 10 g was homogenized with 90 mL of sterile Ringer’s solution in a stomacher. For the determination of each type of microbial growth, a volume (100 µL or 1000 µL) of at least two appropriate serial decimal dilutions (prepared in Ringer’s solution) was plated on the appropriate agar plate substrate; total viable counts (TVCs) were determined using spread plate methodology on tryptic glucose yeast agar (Biolife, IT) after aerobic incubation at 25 °C for 72 h.

### 2.5. Shelf-Life Calculation

Non-pre-treated, OD-treated, and PEF-pre-treated–OD-treated spinach leaves processed at appropriate treatment conditions selected from previous experiments were stored at different temperatures to determine their shelf life. One hundred grams (approximately) of each product was packed into polyethylene-polypropylene sachets (15 × 15 cm) and stored in the dark at constant temperatures between 4 °C and 20 °C (4, 8, 12 °C for untreated samples and 4, 12, 20 °C for OD- and PEF–OD-treated samples). At regular time intervals, samples were taken and analyzed for microbial growth (total aerobic viable counts (*TVC*s)) according to Dermesonlouoglou et al. (2018) [13] and sensory properties.

The evolution of microbial growth at each temperature was modeled by the Gompertz model (Equation (5)) [38]:

(5) $$\log\left(N\right)=logN_{0}+{logN}_{m}e^{-e^{\frac{\mu_{m}e}{{logN}_{m}}\left(\lambda -t\right)+1}}$$

where *log(N)* is the microbial load of the product per gram at time *t*, *log(N*_0_*)* is the initial microbial load, *log(N_m_)* is the maximum difference in microbial load compared to the initial load (such that *log(N*_0_*)* + *log(N_m_)* yields the final microbial load of the product), *μ_m_* is the exponential growth rate, and *λ* is the lag phase. Determination of the model parameters enables the prediction of shelf life as well as secondary shelf-life modeling by expressing the parameters as a function of storage temperature.

The dependence of the overall sensory acceptance on storage temperature is described by the following zero-order equation (Equation (6)):

(6) $$S\left(t\right)=S_{0}\pm k_{s}t$$

where *S* is the sensory score at time *t*, *S*_0_ is the sensory score at time zero, and *k_s_* is the rate of score deterioration. The sign of *k_s_* denotes the direction in which the deterioration occurs: if the score of an attribute increases with storage time, the sign is positive, whereas when the score decreases with time, the sign is negative.

The dependence of the relevant model parameters for both primary models (Equations (5) and (6)) on storage temperature (parameters *k_s_* and *µ_m_*) was mathematically modeled using the Arrhenius equation (Equation (7)):

(7) $$k=k_{Tref}\exp\left(-\frac{E_{a}}{R}\left(\frac{1}{T}-\frac{1}{T_{ref}}\right)\right)$$

where *E_a_* is the activation energy of the parameter *k*, *k_Tref_* is the deterioration rate at the reference temperature *T_ref_*, and *R* is the universal gas constant.

The shelf life of each studied product, determined by the overall sensory acceptance as a function of storage temperature, can be calculated as follows:

(8) $$SL_{s}=\frac{S_{0}-S_{L}}{k_{s_{Tref}}\exp\left(-\frac{E_{a_{s}}}{R}\left(\frac{1}{T}-\frac{1}{T_{ref}}\right)\right)}$$

where *S_L_* is the acceptance limit of the sensory parameter *S*. Using this composite equation, the shelf life of the product can be predicted at various storage temperatures, even at variable temperature profiles common for temperature abuse during real conditions of storage and transport.

Respectively, the shelf life based on total microbial growth is calculated as follows:

(9) $$SL_{TVC}=\frac{logN_{l}-logN_{0}}{\mu_{m}(T)}+\lambda (T)$$

where $logN_{l}$ is the acceptable maximum microbial load limit of the product; $logN_{0}$ is the initial microbial load of the product; and $\mu_{m}(T)$ and λ(T) are the dependence of the rate constant and the lag phase parameter on storage temperature, determined through the Arrhenius equation.

Microbial analysis was carried out before sensorial analysis. Following this experimental procedure, it was ensured that no samples with a microbial load higher than the maximum allowed limit (7.5 *logCFU*/g) were consumed by the panelists. The sensorial characteristics of the samples with a microbial load higher than 7.5 *logCFU*/g (which were not consumed) were only evaluated via optical and smelling observation.

### 2.6. Statistical Analysis

Results were presented as three replicates’ average ± standard deviation. Factorial analysis of variance (Factorial ANOVA) was employed to estimate the main interaction effects of the factors. Duncan’s multiple range test was employed as a post hoc analysis for the separation of means with significant differences (*p* < 0.05) (Statistica 7.0, StatSoft, Hamburg, Germany). For all the mathematical regressions, the IBM SPSS Statistics Version 19 software package (IBM Corporation, Armonk, NY, USA) was used, and R^2^ and standard errors of model parameters were calculated.

## 3. Results

### 3.1. Electric Field Strength Selection for Spinach Pre-Treatment

**Figure 1:**
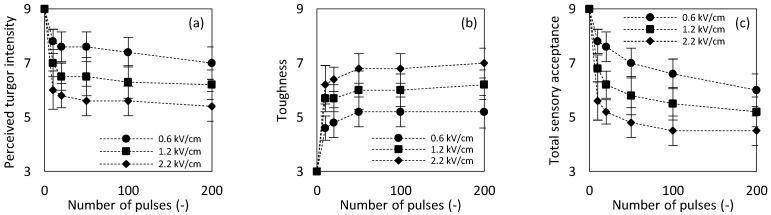
Dependence of (**a**) perceived turgor intensity, (**b**) toughness, and (**c**) total sensory acceptance of spinach on the number of pulses after PEF treatment at 0.6, 1.2, and 2.2 kV/cm. Markers indicate experimental data points.

The pulsed electric field (PEF) treatment significantly affected the sensory characteristics of the spinach leaves. For the application of PEF as a pre-treatment of osmotic dehydration (OD), a wide range of conditions (0.6–2.2 kV/cm, 0–200 pulses) were studied to achieve an effective treatment without product over-processing. The dependence of spinach’s perceived turgor intensity, toughness, and total sensory acceptance on the number of pulses at different electric fields is presented in Figure 1. The increase in electric field strength from 0.6 to 2.2 kV/cm led to a reduction in perceived turgor intensity, while a significant increase in toughness was observed. According to the panel, the PEF treatment at 1.2 and 2.2 kV/cm gave an undesirable chewy sensation that was responsible for the significantly lower values of the total sensory acceptance score. As a result, an electric field of 0.6 kV/cm was selected for further pre-treatment of spinach since the reduction in turgor revealed effective electroporation while the total sensory acceptance score remained higher than 5 for up to 200 pulses.

### 3.2. Selection of Osmotic Medium Formulation and PEF Treatment Conditions for Spinach

**Figure 2:**
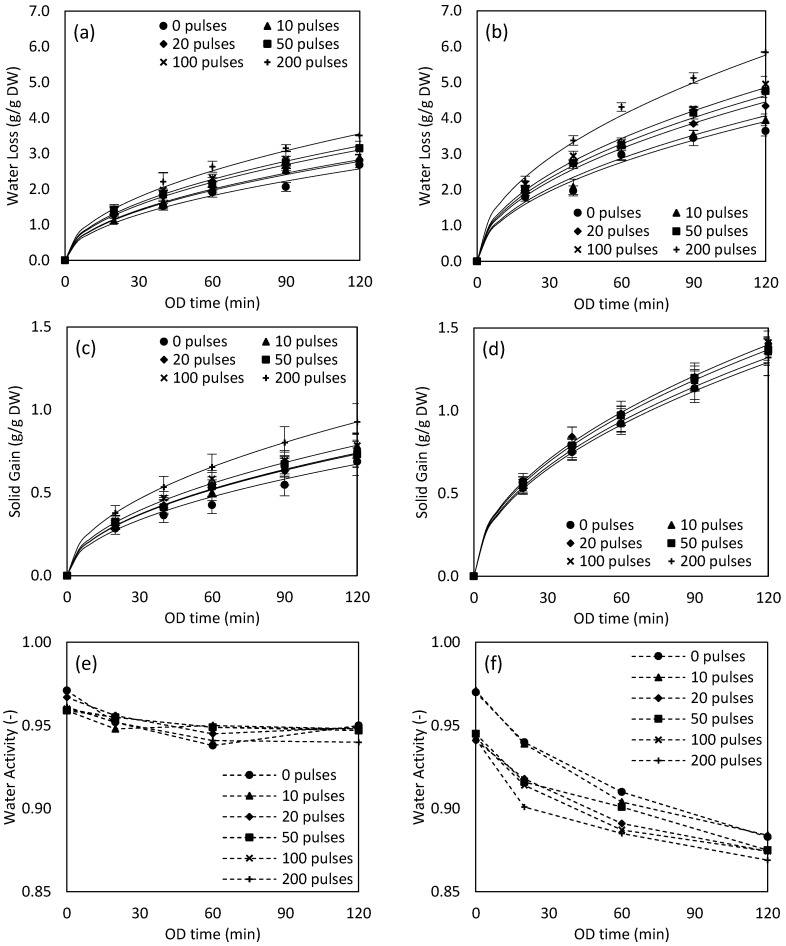
Evolution of water loss (WL) * of spinach samples during OD at (**a**) 50% *w/w* and (**b**) 60% *w/w* glycerol, solid gain (SG) * at (**c**) 50% *w/w* and (**d**) 60% *w/w* glycerol, and water activity (*a_w_*) at (**e**) 50% *w/w* and (**f**) 60% *w/w* glycerol. markers indicate experimental data points. Straight lines correspond to the fitting of the penetration model (Equation (3)) to the experimental data.

The results for the OD of the untreated and OD-treated spinach samples at different glycerol contents in terms of water activity, water loss, and solid gain are presented in Figure 2. It was observed that the dehydration of the spinach leaves was accelerated with the increase in glycerol content, both in terms of decreased water activity and water loss.

The increase in dehydration effectiveness at a higher glycerol content was also reflected in the increase in the water loss and solid gain constants (*k_WL_* and *k_SG_*, respectively), as calculated by the fitting of penetration model Equation (3) to the experimental data presented in Table 1. The significant increase in water loss and solid gain rate, combined with the significant decrease in water activity during OD (0.971 at 0 min to 0.884 at 120 min) for a glycerol content of 60%, led to the selection of the specific medium formulation for the dehydration of spinach leaves. These results agree with Dermesonlouoglou and Giannakourou (2019) [39], who reported that, at a glycerol concentration of 60% *w/w*, both the diffusion of water and the solids were significantly enhanced during the OD of peach.

Regarding the PEF pre-treatment of spinach, the increase in the number of pulses from 0 to 200 led to a significant increase in water loss up to 60.4% after 120 min of OD at 60% glycerol, with a simultaneous increase in *k*_WL_ from 0.357 to 0.gwatergDW·s12(Table 2). The solid gain did not present a significant dependence on the pulse number since the major parameters of SG were the glycerol content and the OD time. The effect of PEF pre-treatment on WL increase during OD corresponds to a significant reduction in water activity, as presented in Figure 2e. Water activity values below 0.91 are commonly adopted as minimum values where bacterial proliferation is significantly reduced [24]. For untreated spinach, an OD time longer than 60 min was required for *a_w_* to drop below 0.9. However, a PEF treatment of 20 pulses led to an *a_w_* reduction to 0.891 compared to 0.910 in the untreated sample after 60 min of OD at 60% glycerol. Further increases in the number of pulses did not lead to a significant *a_w_* reduction after 60 min of OD (0.891 and 0.885 for 20 and 200 pulses, respectively). The condition of 20 pulses at 0.6 kV/cm was the less energy-consuming condition that combined an overall sensory acceptance score of spinach higher than 7 (Figure 1c) with a drop in *a_w_* below 0.9 after 60 min of OD. For these reasons, this PEF condition was chosen as a pre-treatment for OD improvement and to study the extension of the OD-dehydrated product’s shelf life. The positive impact of PEF on the duration of dehydration (reduced time of drying or osmotic dehydration), leading to energy savings and increases in productivity, has been reported [14,25,40]. The PEF treatment has been shown to affect the porosity of both the cell membrane and the cell walls of plant tissues. This increase in permeability is the main driving force behind the observed increase in water transport rates and is relevant to any process that involves mass transfer to and from the treated plant material. This effect is well correlated with the observations made during the preliminary experiments, where a significant loss of turgor and an increase in leaf toughness were reported. Both of these effects are manifestations of intracellular water loss through the permeabilized cell wall as caused by the PEF treatment.

### 3.3. Shelf-Life Calculation of Non- and Pre-Treated Spinach Samples

#### 3.3.1. Evolution of Microbial Load in Non-, OD-Treated, and PEF-Pre-Treated–OD-Treated Spinach Samples

The shelf life of freshly cut leafy salads is the outcome of combined microbiological-dependent and -independent changes during storage, which may represent a vector of quality and spoilage indicators. A complex indigenous spoilage flora comprises *Pseudomonas* spp., lactic acid bacteria, Enterobacteriaceae, and yeasts–molds. Other possible causes of quality degradation include browning and enzymatic softening, which may also be partially attributed to enzymes released by microorganisms [37].

**Figure 3:**
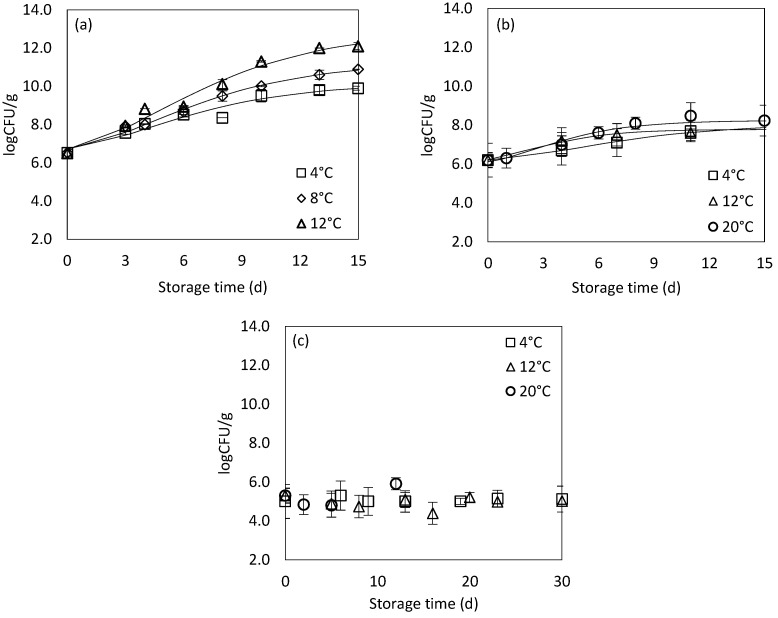
Evolution of microbial load (total viable count) at different storage temperatures for (**a**) untreated, (**b**) OD-treated, and (**c**) PEF-OD-treated spinach samples. Markers indicate experimental data points. Straight lines represent fitting of the Gompertz model (Equation (5)) to the experimental data.

Leafy salads are increasingly being sold as ready-to-eat (RTE) products. As RTE food products are not going to be further processed by consumers, they must be safe to eat within a stated timeframe. The standard methodology for assessing the microbiology of a product is defined in Commission Regulation 2073/2005 (2006) [41], where the specific ISO method for testing, referred to as Aerobic Colony Count, is often used; thresholds vary for what is classed as not acceptable but are usually in the range 10^5^–10^7^ colony forming units per gram (cfu/g) [42,43]. Values in excess of this figure suggest the microbial flora is considered to be from one predominant organism [42]. There are three microorganisms that have specific regulations pertaining to the safety of leafy salads. These are E. coli 0157:H7, Listeria monocytogenes, and Salmonella. Salmonella and Listeria monocytogenes have regulations that are in place while the product is on the shelves [41,42,43]. These organisms are controlled via the washing/sanitization step and Good Hygiene Practices [44]. E. coli is considered a hygienic indicator during the manufacturing stage [45]. We primarily focused on quality changes incurred by spoilage, microbial growth, and/or enzymatic activity. The dependence of microbial growth (total viable counts) on storage time for different storage temperatures is presented in Figure 3 for untreated, OD-treated, and PEF-OD-treated spinach samples. (Growth data for *Pseudomonas* spp., yeasts and molds, and *Enterobacteriaceae* are provided in Supplementary Files; Supplementary Figures S1–S3.)

Microbial growth in untreated spinach exhibited an increased rate as the storage temperature increased from 4 °C up to 12 °C (Figure 3a). A storage temperature increase was found to affect all types of microbial flora determined. Total viable counts start at approximately 6.5 *logCFU*/g and reach values exceeding 10 *logCFU*/g. It is well established that leafy vegetables exhibit an extremely high initial microbial load, with *Pseudomonas* spp. and *Enterobacteriaceae* dominating the microflora [46,47]. It was reported that a critical population (i.e., 8.0–9.0 *logCFU/g*) of the dominant microorganism (Specific Spoilage Organism) determines the end of the microbial shelf life of fresh-cut salads [37]. This is clearly demonstrated in our results for the untreated and OD-treated spinach samples (Supplementary Files). However, a significant shift in the temperature dependence of microbial growth was observed for all the studied substrates, with storage temperatures for OD-treated spinach nearing room temperature (20 °C). Unlike the measurable growth observed for both untreated and OD-treated spinach, in the case of the PEF-OD-treated samples, the microbial load appeared stagnant and even decreased with storage time at all the studied storage temperatures (Figure 3c). No clearly discernible trend was observed for the evolution of total viable counts. These observations may be explained by several factors. The approach proposed in our study resulted in the production of products with a low water activity value (*a_w_* = 0.884). pH values ranged from 4.3 (PEF-OD-treated) to 5.6 (untreated). pH increased up to 7.7 for the untreated samples, which correlated with the growth of *Pseudomonas*. A similar trend was observed for the OD-treated samples, but the pH values obtained did not exceed 5.5 due to the intense uptake of acetic acid in the OD solution. Contrarily, the PEF-OD-treated samples exhibited a stable, relatively low pH value of around 4.3 during the whole storage period due to the intensified mass transfer during OD caused by electroporation. This created even more unfavorable conditions for microbial growth. It is possible that what was observed was in fact an extended lag phase. Nevertheless, the experiment ceased due to the unacceptable deterioration of other quality parameters, and microbial counts were not measured further. Dermesonlouoglou et al. (2016a, 2020) reported similar behavior in OD-processed fruit samples (pumpkin, strawberry) [48,49]. The osmotically dehydrated samples presented low values (up to 3–4 *logCFU*, the average value), initially and during refrigerated storage, related to the low water activity (<0.91) and low pH (3.6–3.7) values of the final OD samples. In these cases, the shelf life of the OD-treated samples was determined by sensory rejection (overall visual appearance, texture and color degradation, off-odor development).

The total viable counts for the untreated and OD-treated samples were mathematically modeled using the Gompertz model for microbial growth (Equation (5)). The model parameters are given in the table below (Table 3). The dependence of the exponential rate constant *μ_m_* on storage temperature was modeled using the Arrhenius equation with the aim of determining the activation energy (*E_a_*).

Comparing the model parameters for the two treatments (untreated and OD-treated), it becomes evident that a significant shift in storage temperature dependence occurs. At chilled storage (4–12 °C), the exponential growth rate *μ_m_* of the total microflora in the OD-treated samples decreased by 50%. This inhibition is attributed to several factors, such as a reduction in water activity and the uptake of inhibitory compounds from the osmotic solution (e.g., acetic acid and sodium chloride). Nevertheless, even though microbial growth was not as prominent as in the untreated samples, it could be mathematically modeled. The same could not be observed in the PEF-OD-treated samples, where no trend in microbial growth was observed. Finally, neither treatment led to a satisfactory calculation of the lag phase parameter (*λ*).

#### 3.3.2. Evolution of Sensory Characteristics for Untreated, OD-Treated, and PEF-OD-Treated Spinach Samples

**Figure 4:**
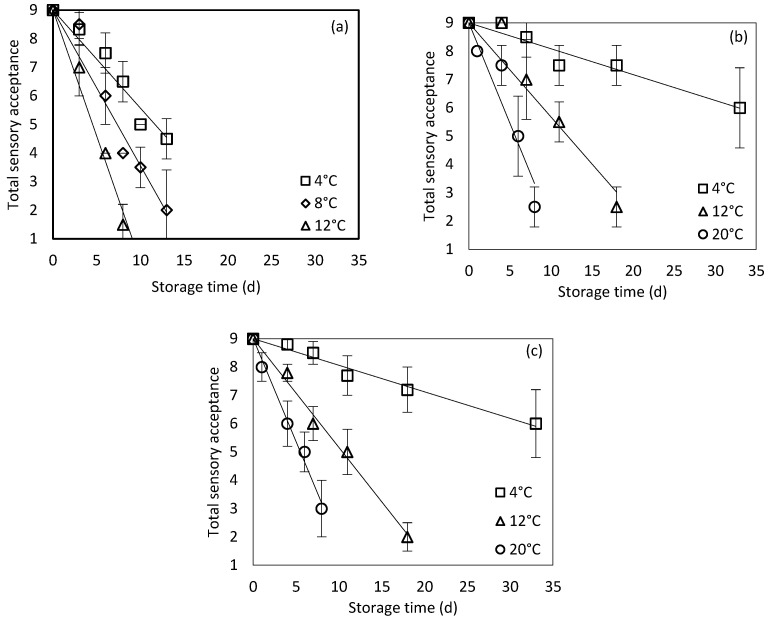
Evolution of the overall sensory acceptance during storage at various temperatures (4, 8, 12, and 20 °C) for (**a**) untreated, (**b**) OD-treated, and (**c**) PEF-OD-treated spinach leaves. Markers indicate experimental data points. Straight lines correspond to the fitting of Equation (6) to the experimental data.

The evolution of total sensory acceptance with storage time for all the storage temperatures studied for the untreated, OD-treated, and PEF-OD-treated spinach samples is presented in Figure 4.

All the tested sensory characteristics exhibited deterioration over storage time. The effect of storage temperature did not appear to significantly alter the course of this deterioration, probably due to the relatively short storage periods studied and the narrow temperature range. The untreated spinach leaves faded in color during storage (increase in perceived luminosity and loss of green color), while turgor and tear resistance were lost. The panelists reported an increase in wilting as storage time progressed and also reported the emergence of a bitter aftertaste. It was observed that the main factor that dominated the deterioration of the untreated spinach leaves was the development of off-flavors and off-odors. This was correlated with microbial growth, as discussed above, and is a common occurrence in leafy vegetables. Although the other characteristics were not significantly affected by the different storage temperatures, off-odor development and overall acceptance exhibited a clear temperature dependence, which further validates their connection to microbial spoilage.

Crucial to the attractive appearance of spinach is its green color, which depends on the amounts of chlorophylls, the ratio of chlorophyll a to chlorophyll b, and, of course, their degradation towards pheophytins. The intensity of the green color undergoes a discernible alteration during storage. This change may be indicative of chlorophyll degradation or other color-degrading processes such as enzymatic browning. The OD-treated leaves appeared to experience a notable decline in green color intensity since, during OD, there was a loss of pigments from the product into the osmotic solution. However, this was not as prominent as for the untreated samples since the constituents of the OD solution, combined with the reduction in product water content, may have acted protectively towards the chlorophylls [50]. In contrast, perceived luminosity remained relatively constant over the storage period, with score values ranging between 4 and 5, as opposed to the untreated samples, where significant fading was observed (perceived luminosity increased from 1 to 5) (see Supplementary Figure S4). The lack of significant variation in luminosity implies that the treatment and storage conditions may not significantly influence the overall brightness or visual perception of the spinach leaves. Enzyme activity can also produce color changes by changing the surface’s ability to reflect light, which is caused by the air removed from the vegetable tissue and its replacement with water or cell juice as a result of cell membrane disruption [51]. Enzymatic reactions are not ruled out and are still possible because neither the mild PEF treatment conditions used nor the mild dehydration effects of OD are sufficient to inhibit them. The results also suggest a more pronounced loss of turgor compared to the untreated sample, which is also somewhat expected since water removal from plant cells unavoidably leads to loss of cell turgor. However, elasticity did not exhibit a significant change with storage time, implying a certain resilience in the mechanical properties of the treated leaves, which was not as prominent as for the untreated samples. However, this preservation of elasticity may suggest the presence of a degree of gumminess imparted by the OD treatment. Tear resistance remained relatively stable during storage, indicating that the treated spinach leaves maintained a consistent level of resistance against tearing. This stability is an important consideration for the overall durability and handling of the leaves. In this study, the most prominent factor contributing to sensory deterioration seems to have been the development of off-flavors. The increasing intensity of off-flavors over time suggests a degradation in taste and aroma, potentially linked to biochemical changes during storage but also to microbial growth.

Overall acceptance encompasses the panelists’ general opinion about the examined samples and is usually a good indicator of the evolution of a product’s quality. In Figure 4, the overall sensory acceptance scores of the untreated, OD-treated, and PEF-OD-treated spinach samples are representatively presented. By fitting the zero-order kinetic model to the experimental data, it was possible to determine the rates of sensory deterioration at each storage temperature (*k_s_*). The results of the fitting for the untreated, OD-treated, and PEF-OD-treated samples are presented in Table 4.

#### 3.3.3. Evolution of Selected Quality Indices of Untreated, OD-Treated, and PEF-OD-Treated Spinach Samples

**Figure 5:**
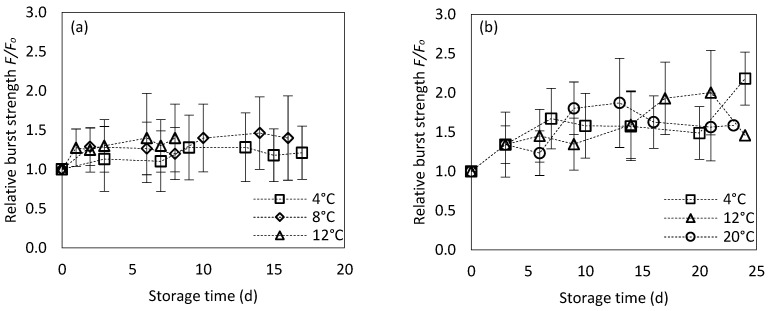

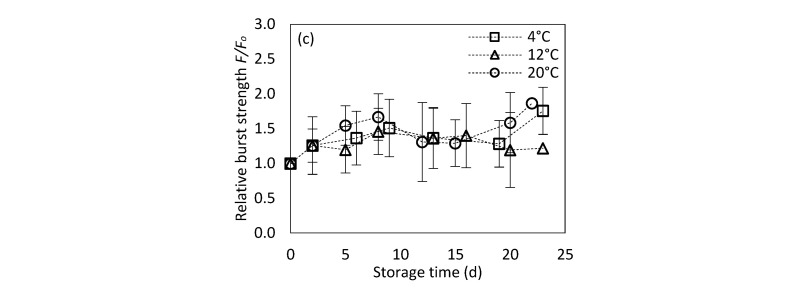
Evolution of the relative burst strength of (**a**) untreated, (**b**) OD-treated, and (**c**) PEF-OD-treated spinach samples. Markers indicate experimental data points.

The evolution of the relative burst strength of the untreated, OD-treated, and PEF-OD-treated spinach samples at different storage temperatures is presented in Figure 5. For all treatments, it was observed that the burst strength of the spinach leaves increased with storage time. This effect was more pronounced when an OD treatment was performed, with the relative burst strength of the treated leaves almost doubling after 25 days of storage. Macroscopically, this can manifest as an increased elasticity and gumminess of the leaves, which was confirmed by the sensory evaluation of the samples. Due to the loss of water during storage (untreated samples) or dehydration (OD-treated samples), the fibers in the plant tissue became compacted, leading to an increased mechanical strength of the tissue [52]. Additionally, no noticeable differences in burst strength were observed between the treatments. This is a positive result since OD and PEF did not lead to texture degradation in the final product.

**Figure 6:**
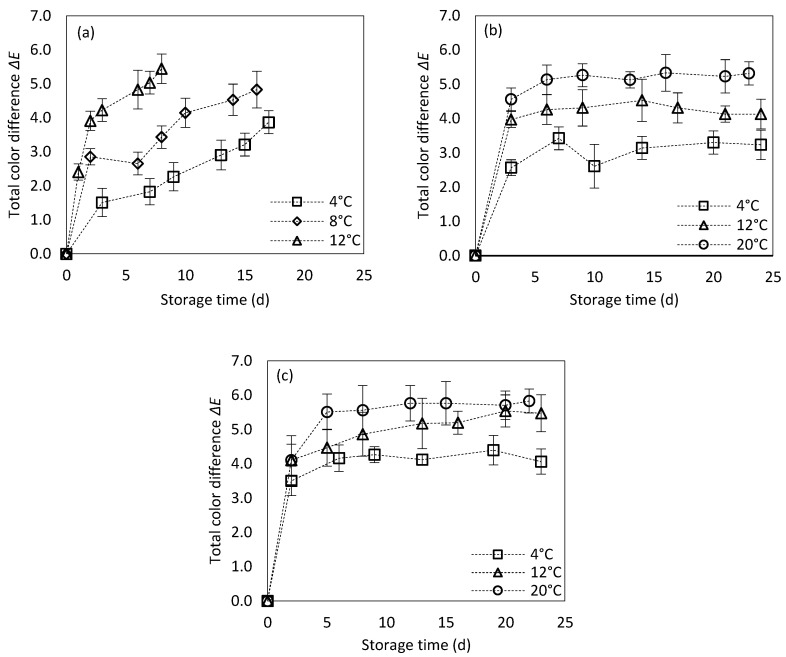
Evolution of the total color difference ΔΕ calculated with the untreated sample at time 0 as a reference for (**a**) untreated, (**b**) OD-treated, and (**c**) PEF-OD-treated spinach samples with respect to storage time at all storage temperatures studied (4, 8, 12, and 20 °C). Markers indicate experimental data points.

The evolution of the total color difference (ΔΕ) of the spinach samples for all treatments with respect to storage time is presented in Figure 6. For all the treatments studied, a temperature-dependent color deterioration was observed in the spinach leaves, which was more pronounced in the OD-treated and PEF-OD-treated samples (Figure 6b,c).

### 3.4. Shelf-Life Modeling of Treated Spinach

Based on the results obtained from both the sensory and the microbial growth mathematical modelings, it was possible to determine the shelf life based on both parameters. The results of this calculation are presented in Table 5. The shelf life of the treated spinach samples was mainly determined by microbial spoilage, with a maximum allowed limit of 7.5 *logCFU*/g for temperatures up to 12 °C. The determination of the shelf life based on sensory deterioration at these temperatures (with a limit score of 5) led to an overestimation of the shelf life by several days, especially at lower temperatures, indicating that the microbial growth does not necessarily lead to sensory unacceptability of the treated product. This trend was reversed for OD-treated spinach at 20 °C, where other biochemical reactions dominate and cause the shelf life to be defined by sensory deterioration.

Our results verify the extremely short shelf life of the untreated spinach leaves at chilled storage, which was short of 5 days. All treatments involving OD led to a significant shelf-life extension compared to the untreated samples at chilled storage from 7.5 days (OD-treated) up to over 30 days (PEF-OD-treated), underlining the effectiveness of osmotic dehydration to extend the shelf life of even the most perishable and highly contaminated spinach. This extension had a significant effect on the temperature tolerance of the samples, with a shelf life of approximately 6 days at 20 °C for the OD-treated samples, comparable to the values obtained at 4 °C for the untreated samples.

The implications of such an effective processing outcome are expectedly significant from an economic perspective. Although a concrete statement would require an in-depth cost analysis, which is not within the scope of this work, there are several points that can speak in favor of a potentially successful transfer of the whole process to an industrial, production-ready, and economically sensible scale.

First, OD requires very simple and straightforward equipment that can be as simple as a stirred tank. In the case of spinach, no external heating is required, making the required equipment even simpler and more affordable. Secondly, significant savings can be achieved by replenishing, reusing, pasteurizing, and condensing the spent osmotic solution after each cycle. This is a well-established approach in industrial OD processing. Thirdly, pulsed electric fields are a relatively mature technology, and there are several continuous-processing industrial devices available on the market covering a wide range of required productivities. Finally, the significant shelf-life extension of highly perishable spinach is expected to lead to food waste reduction and energy savings during storage and also enable food retailers to distribute spinach-based products to more distant markets, which might have hitherto been impossible.

## 4. Conclusions

This study aimed to assess the feasibility of pulsed electric fields (PEFs) and osmotic dehydration (OD) for enhancing the processing of spinach leaves, with a focus on extending shelf life and improving quality attributes. Both treatments demonstrated their ability to extend the shelf life of spinach by up to four-fold, or, equivalently, by significantly raising the required storage temperature to achieve the same shelf life. Treated spinach leaves can serve as convenient chilled snacks or versatile ingredients in various food formulations, such as ready-to-eat salads. This research presents opportunities to enhance fresh vegetable or salad processing techniques and diversify their culinary uses, aligning with consumer preferences for high-quality, nutritious options with reduced energy consumption.

## Figures and Tables

**Table 1:** Different osmotic dehydration (OD) and pulsed electric field (PEF) treatment variables and their levels.

Treatment	Experimental Conditions			
Pulsed Electric Fields (PEFs)	Electric (kV/cm)	Pulses (p)	Temperature (°C)	Other
	0.6, 1.2, 2.2	0, 10, 20, 50, 100, 200	25	Pulse width: 15 μ; pulse frequency: 20 Hz
Osmotic Dehydration (OD)	Glycerol concentration (%)	Temperature (°C)	Time (min)	Other
	50, 60	25	0, 20, 40, 60, 90, 120	1.5% calcium chloride, 1% sodium chloride, 2.5% vinegar, 0.05% *w/w* sodium sulfite Solid-to-liquid ratio 1:20

**Table 2:** Water loss and solid gain constants for different PEF pre-treatment and glycerol contents as calculated from the penetration model (Equation (3)) fitting to the experimental data.

PEF Pre-Treatment	Water Loss Constant kWLgwatergDW·s12				Solid Gain Constant kSGgsolidsgDW·s12			
	50% *w/w* Glycerol	R^2^	60% *w/w* Glycerol	R^2^	50% *w/w* Glycerol	R^2^	60% *w/w* Glycerol	R^2^
0 pulses	0.235 ± 0.010 ^a^	0.930	0.357 ± 0.012 ^e^	0.930	0.061 ± 0.005 ^a^	0.912	0.118 ± 0.008 ^c^	0.914
10 pulses	0.254 ± 0.016 ^ab^	0.920	0.371 ± 0.010 ^ef^	0.927	0.068 ± 0.004 ^a^	0.911	0.118 ± 0.009 ^c^	0.934
20 pulses	0.258 ± 0.008 ^ab^	0.933	0.407 ± 0.018 ^fg^	0.934	0.068 ± 0.006 ^a^	0.933	0.128 ± 0.008 ^c^	0.918
50 pulses	0.294 ± 0.016 ^c^	0.925	0.423 ± 0.010 ^gh^	0.922	0.070 ± 0.005 ^a^	0.925	0.125 ± 0.009 ^c^	0.927
100 pulses	0.283 ± 0.014 ^bc^	0.934	0.443 ± 0.020 ^h^	0.925	0.072 ± 0.004 ^a^	0.928	0.126 ± 0.011 ^c^	0.941
200 pulses	0.325 ± 0.013 ^d^	0.928	0.526 ± 0.028 ^i^	0.921	0.085 ± 0.006 ^b^	0.923	0.121 ± 0.010 ^c^	0.911

Different superscript letters indicate significant differences between means *k*_WL_ and *k*_SG_ ± standard deviation as calculated by Duncan’s multiple range test for a significance level of *p* = 0.05.

**Table 3:** Key Gompertz model parameters for microbial growth (total viable counts) for untreated and OD-treated spinach samples. The dependence of the rate constant *μ_m_* on storage temperature was modeled with the Arrhenius equation.

Storage Temperature (°C)	$\mu_{m}(d^{-1})$		$logN_{o}\left(\frac{logCFU}{g}\right)$		$logN_{m}\left(\frac{logCFU}{g}\right)$		R^2^	
	Untreated	OD-Treated	Untreated	OD-Treated	Untreated	OD-Treated	Untreated	OD-Treated
4	0.32 ± 0.02 ^d^	0.16 ± 0.01 ^a^	6.48 ± 0.10 ^a^	6.06 ± 0.06 ^d^	3.70 ± 0.06 ^c^	2.02 ± 0.07 ^e^	0.979	0.994
8	0.41 ± 0.02 ^e^	-	6.37 ± 0.09 ^ab^	-	4.91 ± 0.05 ^b^	-	0.986	-
12	0.52 ± 0.03 ^f^	0.26 ± 0.01 ^b^	6.26 ± 0.05 ^c^	6.11 ± 0.04 ^d^	6.69 ± 0.06 ^a^	1.67 ± 0.08 ^f^	0.982	0.959
20	-	0.31 ± 0.02 ^c^	-	5.97 ± 0.10 ^d^	-	2.27 ± 0.05 ^d^	-	0.968
$E_{a}\left(\frac{kJ}{mol}\right)$	−39.9 ± 4.1	−28.8 ± 3.5	-	-	-	-	-	-
	R^2^ = 0.997	R^2^ = 0.953						

Different superscript letters indicate significant differences between means *μ_m_*, *logN_o_*, and *logN_m_* ± standard deviation as calculated by Duncan’s multiple range test for a significance level of *p* = 0.05.

**Table 4:** Rate constants of sensory deterioration (overall sensory acceptance) for untreated, OD-treated, and PEF-OD-treated spinach samples for all storage temperatures studied. The dependence of the rate constants on storage temperature was mathematically modeled using the Arrhenius equation, and the values of the activation energy are given in the last row of this table.

Storage Temperature (°C)	Non-Pre-Treated Spinach		OD-Treated Spinach		PEF-Pre-Treated–OD-Treated Spinach	
	$\mathrm{Sensory}\;\mathrm{Deterioration}\;\mathrm{Rate}\;k_{s}(d^{-1})$	R^2^	$\mathrm{Sensory}\;\mathrm{Deterioration}\;\mathrm{Rate}\;k_{s}(d^{-1})$	R^2^	$\mathrm{Sensory}\;\mathrm{Deterioration}\;\mathrm{Rate}\;k_{s}(d^{-1})$	R^2^
4	0.375 ± 0.015 ^b^	0.959	0.094 ± 0.002 ^a^	0.939	0.090 ± 0.003 ^a^	0.977
8	0.588 ± 0.042 ^c^	0.963	-		-	
12	0.935 ± 0.063 ^e^	0.984	0.387 ± 0.011 ^b^	0.957	0.367 ± 0.027 ^b^	0.991
20	-		0.750 ± 0.041 ^d^	0.910	0.722 ± 0.038 ^d^	0.990
$E_{a}\;\left(\frac{kJ}{mol}\right)$	−74.9 ± 6.1	0.997	−79.8 ± 7.8	0.964	−85.2 ± 9.2	0.967

Different superscript letters indicate significant differences between mean *k_s_* ± standard deviation as calculated by Duncan’s multiple range test for a significance level of *p* = 0.05.

**Table 5:** Determination of untreated, OD-treated, and PEF-OD-treated spinach shelf life based on microbial growth (total viable counts) and sensory deterioration (overall sensory acceptance) for all storage temperatures studied. The bold numbers correspond to the actual sample shelf life based on its corresponding limiting parameter.

Storage Temperature (°C)	Untreated Spinach		OD-Treated Spinach		PEF-OD-Treated Spinach	
	SL Microbial Growth (d)	SL Sensory Deterioration (d)	SL Microbial Growth (d)	SL Sensory Deterioration (d)	SL Microbial Growth (d)	SL Sensory Deterioration (d)
4	**4.7** ± 0.4 ^B^	10.6 ± 0.9 ^b^	**12.2** ± 1.0 ^C^	42.5 ± 2.1 ^d^	-	**38.1** ± 3.2 ^c^
8	**4.0** ± 0.3 ^AB^	6.8 ± 0.5 ^a^	-	-	-	-
12	**3.3** ± 0.3 ^A^	4.2 ± 0.3 ^a^	**7.3** ± 0.5 ^C^	10.3 ± 0.9 ^b^	-	**10.2** ± 1.1 ^b^
20	-	-	6.6 ± 0.4 ^D^	**5.3** ± 0.3 ^a^	-	**5.8** ± 0.5 ^a^

Different superscript letters indicate significant differences between means of shelf life ± standard deviation (capital letters for microbial growth and lowercase letters for sensory deterioration) as calculated by Duncan’s multiple range test for a significance level of *p* < 0.05.

## Data Availability

The original contributions presented in the study are included in the article, further inquiries can be directed to the corresponding author.

## Supplementary Materials

The following supporting information can be downloaded at https://www.mdpi.com/article/10.3390/foods13091410/s1: Figure S1: Evolution of microbial load at different storage temperatures for untreated spinach samples. (a) *Pseudomonas* spp.; (b) yeasts and molds; and (c) Enterobacteriaceae. Markers indicate experimental data points. Dashed lines represent fitting of the Gompertz model to the experimental data. Figure S2: Evolution of microbial load at different storage temperatures for OD-treated spinach samples. (a) *Pseudomonas* spp.; (b) yeasts and molds; and (c) Enterobacteriaceae. Markers indicate experimental data points. Dashed lines represent fitting of the Gompertz model to the experimental data, where applicable. Figure S3: Evolution of microbial load at different storage temperatures for PEF-OD-treated spinach samples. (a) Total viable counts; (b) *Pseudomonas* spp.; (c) yeasts and molds; and (d) Enterobacteriaceae. Figure S4: Evolution of sensory characteristics during storage at various temperatures for untreated spinach leaves: (a) intensity of green color, (b) perceived luminosity, (c) perceived turgor, (d) perceived elasticity, (e) tear resistance, (f) off-flavor intensity. Markers indicate experimental data points. Dashed lines represent fitting of a zero-order kinetic model, where applicable. Figure S5: Evolution of sensory characteristics during storage at various temperatures for OD-treated spinach leaves: (a) intensity of green color, (b) perceived luminosity, (c) perceived turgor, (d) perceived elasticity, (e) tear resistance, (f) off-flavor intensity. Markers indicate experimental data points. Dashed lines represent fitting of a zero-order kinetic model, where applicable.
