# Supplementary material for: Effect of Pulsed Electric Fields on the Shelf Stability and Sensory Acceptability of Osmotically Dehydrated Spinach: A Mathematical Modeling Approach
Source: Foods. 2024 May 3;13(9):1410. doi: 10.3390/foods13091410 (PMC11083339; doi:10.3390/foods13091410)
Supplement: Supplementary file 1 [file foods-13-01410-s001.zip › foods-2941850-supplementary.pdf]

(a) *Pseudomonas* spp.

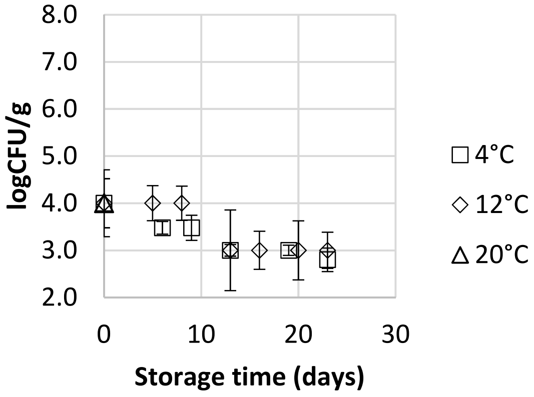

(b) Yeasts and moulds

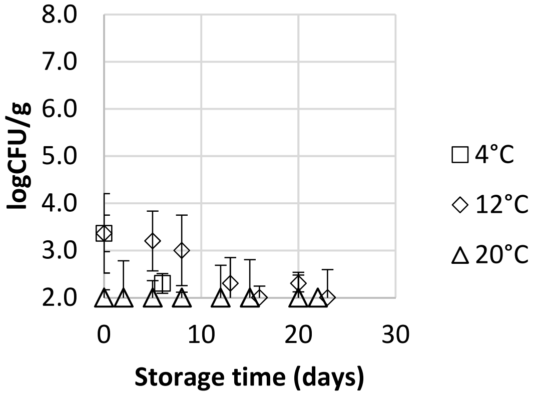

(c) *Enterobacteriaceae*

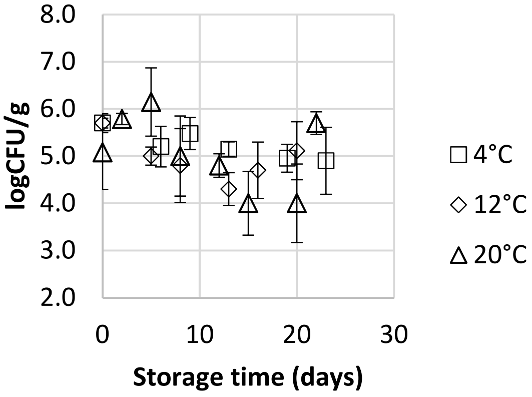

Suppl Figure S1. Evolution of microbial load at different storage temperatures for untreated spinach samples. (a(Pseudomonas spp. (b) yeasts and molds and (c) Enterobacteriaceae. Dashed lines represent fitting of the Gompertz model to the experimental data.*

*Yeasts and molds were enumerated using spread plate methodology on Rose Bengal Chloramphenicol agar (B Enterobacteriaceae spp. were enumerated using pour plate methodology in Violet Red Bile Glucose agar (VRBG, Merck, Darmstadt, Germany) after incubation under a facultatively anaerobic condition at 37 °C for 24 h. iolife, IT) after aerobic incubation at 25 °C for 5 days. Pseudomonas spp. were enumerated using spread plate methodology on Cetrimide agar (CFC, Merck, Darmstadt, Germany) after aerobic incubation at 25 °C for 48 h

(a) *Pseudomonas* spp .

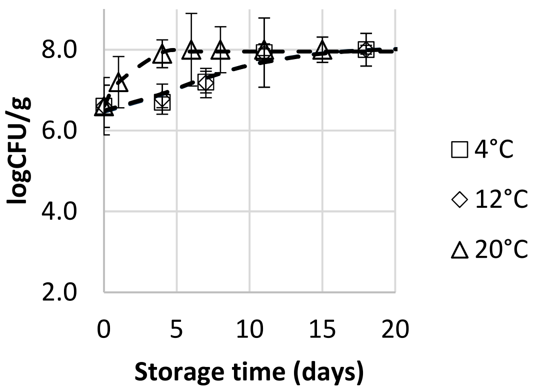

(b) Yeasts and moulds

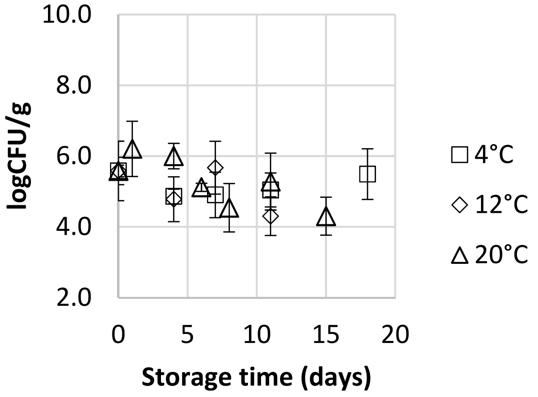

(c) *Enterobacteriaceae*

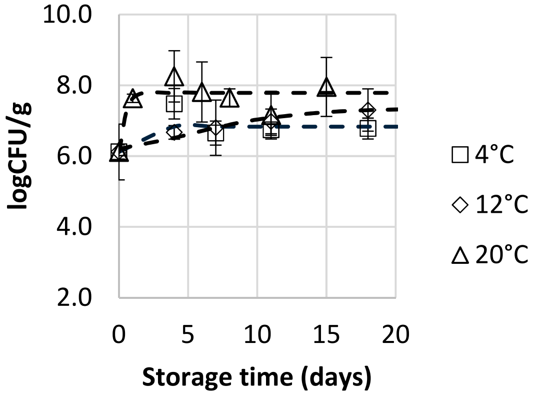

Suppl Figure S2. Evolution of microbial load at different storage temperatures for OD-treated spinach samples. (a) Pseudomonas spp. (b) yeasts and molds and (c) Enterobacteriaceae. Dashed lines represent fitting of the Gompertz model to the experimental data where applicable.

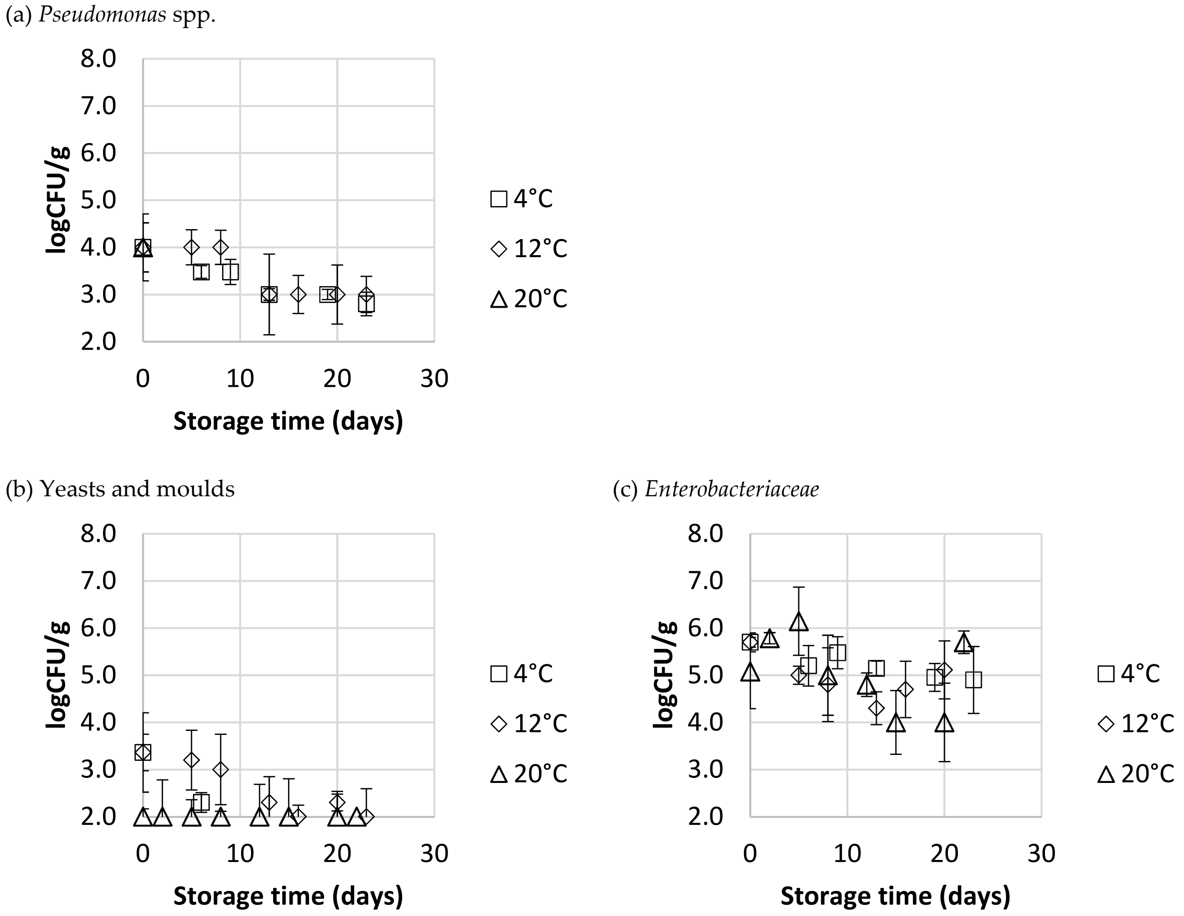

Suppl Figure S3. Evolution of microbial load at different storage temperatures for PEF-OD-treated spinach samples. (a) Total viable counts (b) Pseudomonas spp. (c) yeasts and molds and (d) Enterobacteriaceae.

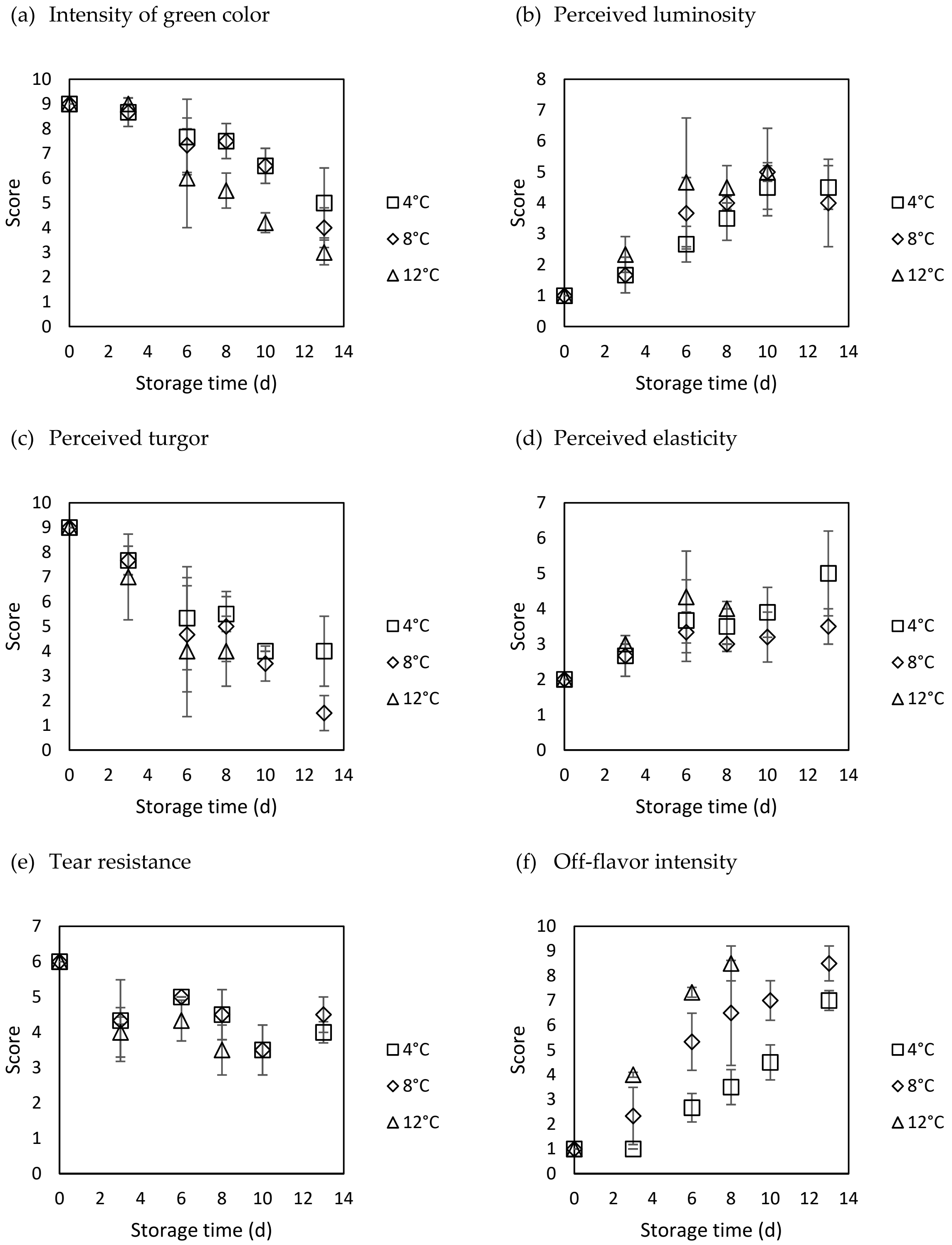

Suppl Figure S4. Evolution of sensory characteristics during storage at various temperatures for untreated spinach leaves: (a) intensity of green color, (b) perceived luminosity, (c) perceived turgor, (d) perceived elasticity, (e) tear resistance, (f) off-flavor intensity. Dashed lines represent fitting of a zero-order kinetic model, where applicable

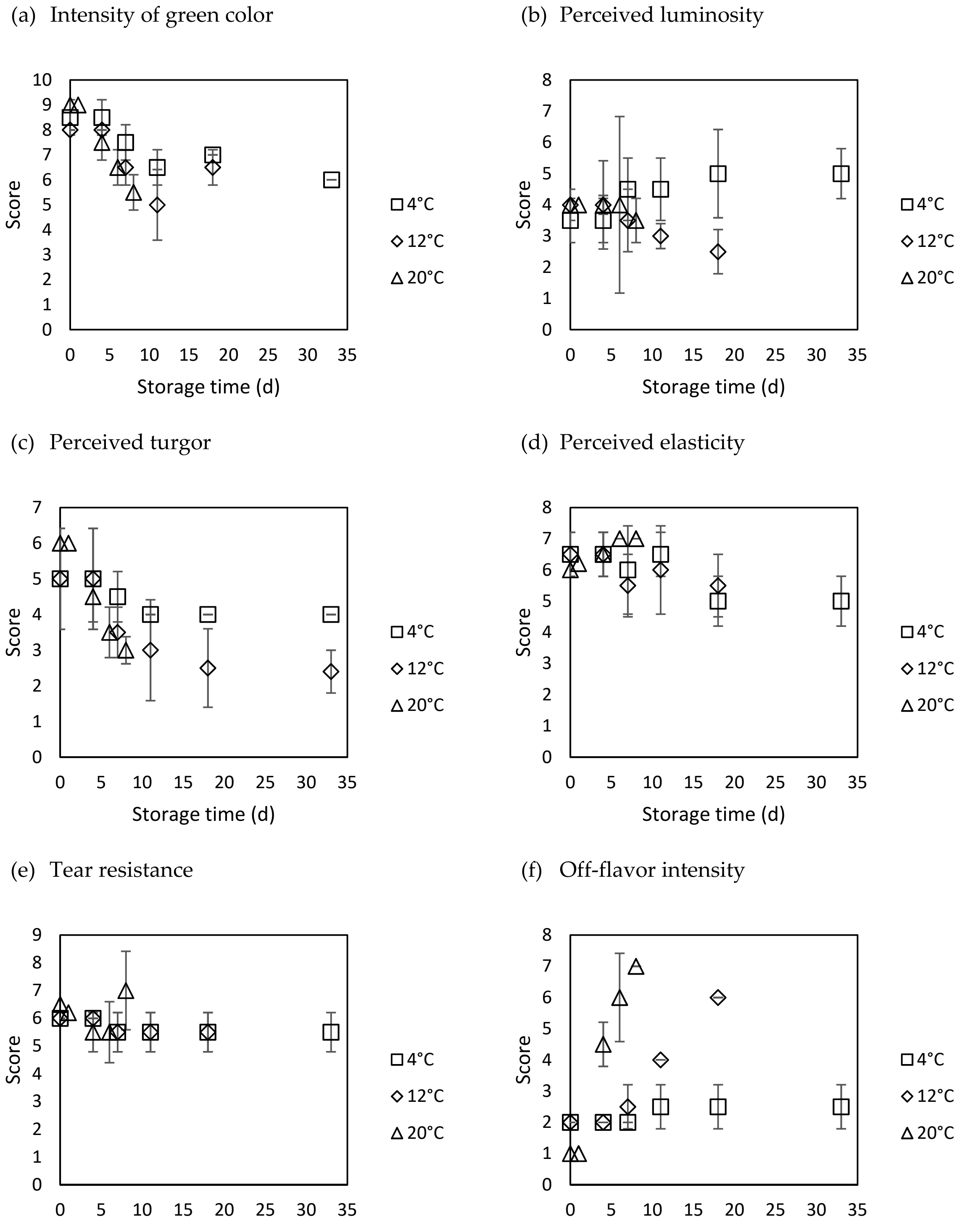

Suppl Figure S5. Evolution of sensory characteristics during storage at various temperatures for OD treated spinach leaves: (a) intensity of green color, (b) perceived luminosity, (c) perceived turgor, (d) perceived elasticity, (e) tear resistance, (f) off-flavor intensity. Dashed lines represent fitting of a zero-order kinetic model, where applicable.
